# The relationship between functional status, physical fitness and cognitive performance in physically active older adults: A pilot study

**DOI:** 10.1371/journal.pone.0194918

**Published:** 2018-04-09

**Authors:** U. Ramnath, L. Rauch, E. V. Lambert, T. L. Kolbe-Alexander

**Affiliations:** 1 Department of Human Biology, Division of Exercise Science and Sports Medicine, Faculty of Health Sciences, University of Cape Town, Cape Town, South Africa; 2 School of Health and Well-being, University of Southern Queensland, Ipswich, Australia; Banner Alzheimer’s Institute, UNITED STATES

## Abstract

**Objective:**

Ageing is associated with a progressive decline in physical function and cognitive performance which could result in a shift from an independent to a more dependent lifestyle. The aim of this research study was to assess the fitness, functional performance and cognitive ability in independently living older South Africans and to determine which fitness parameters and functional performance tests best explain the variance in cognitive function.

**Design:**

Descriptive observational study.

**Participants:**

Older adults with a mean age 71±4.7 years (n = 70; 28 men and 42 women) were recruited. Sixty percent of the sample completed at least secondary schooling and more than two-thirds were taking medication for a chronic medical condition.

**Measurements:**

Self-reported physical activity was assessed using the Yale Physical Activity Survey. Fitness tests included the 6-minute walk test and Bicep Curls. The functional performance tests were; Static and Dynamic balance, Timed Up and Go, Sit to Stand, Grip strength and Functional Reach. The Stroop Task and 6-Item cognitive impairment test were used to measure cognitive performance. Bivariate and multivariate analyses were conducted between performance on the novel cognitive Stroop Task and functional and cognitive tests.

**Results:**

We found significant relationships between the number of correct responses on the Stroop Task and scores on the 6-Item Cognitive Impairment test (-0.520, p < 0.01) and grip strength (r = 0.42, p< 0.01). The number of incorrect responses was inversely associated with functional reach (r = -0.445, p< 0.01). The final regression model included: age, dynamic balance, right arm grip strength and the score on the 6-item cognitive impairment test, and explained 44% of the variance in performance of the Stroop Task.

**Conclusions:**

The results of this study showed that measures of physical function were associated with cognitive performance even in highly functioning older South African adults. Further research is needed to determine the extent to which exercise training can improve functional capacity and the effect on cognitive performance.

## Introduction

The African population is ageing and the projected shift suggests that the number of adults older than 60 years is expected to double over the next 50 years [1]. South Africa has the highest proportion of older persons in southern Africa with approximately 7.6% of the population over the age of 60 years [2]. As life expectancy increases in the older adult population, the prevention of disability and maintenance of functional ability and levels of independence is becoming more important [3].

Physiological changes that occur with ageing include a decrease in muscle mass [4] and a decline in cognitive function [5,6]. Declines in strength and aerobic capacity are associated with declines in functional ability, and ultimately result in decreased levels of independence [7]. Previous research has shown that participation in physical activity declines with age and is associated with obesity and cardiovascular diseases [8]. This contributes to decreased functional ability and increased need for assistance with activities of daily living. Functional ability is defined as the ability to perform activities of daily living independently without any undue pain and fatigue [9]. Functional ability and physical fitness are linked. Physical fitness is defined as a general state of good somatic health that allows an individual the functional capacity to live independently in a safe and independent manner [6]. A decline in function can be observed in a variety of motor abilities due to diminished muscle size, decline in strength, and a reduction in speed which can lead to the decrease in physical power, eventually leading to disability [10]. A decrease in muscle size and strength, both of which influence power, could impact negatively on performance of regular daily activities [9]. Examples of activities where lower body strength is important include: walking, climbing up and down stairs and rising from a seated position.

Physical function is made up of both physiological and cognitive capacities. A decline in function and slower mobility has also been associated with reduced mental speed [11]. Voelcker-Rahage et al. investigated the relationship between physical and motor fitness and cognitive performance. The researchers reported that cardiovascular fitness and muscle strength were both positively associated with cognitive performance in German older adults. In addition, decreased motor fitness was associated with decreased cognitive performance in men and women older than 65 years [11].

A decline in cognitive function could also influence quality of life and independent living of older adults [12]. Ageing is known to be associated with a decrease in cognitive processes such as mental speed, short term memory, reaction time and control skills like planning [3]. Previous studies have shown that a number of factors such as physical activity, education and chronic life stress can influence cognitive function as individuals age [13–15].

A wide variety of well-established test batteries have been used in other research studies to assess the different domains related to a decline in function with ageing. These include assessments such as the Continuous-Scale Physical Functional Performance test [16] and the Senior Fitness Test [17] for functional performance, while other studies have used bespoke test batteries to assess other domains [15]. One example of a bespoke test battery was developed by Williamson and Colleagues, involving the modified Stroop Task to assess cognitive function [15]. Much of the published research in this area has been conducted with individuals who had cognitive impairments or among those living in retirement or assisted-living care facilities [12,18,19]. Other studies have been conducted in individuals who were inactive and had a sedentary lifestyle [17].

Physical fitness, functional ability and cognitive function all play an important role in maintaining the independence and quality of life of older adults. Therefore, the aim of this research was to investigate the relationship between functional ability, fitness and cognitive performance in high functioning, independently-living, older South Africans. A secondary aim was to determine the extent to which physical, cognitive and functional parameters predict performance in a novel cognitive task. We hypothesized that a higher level of physical activity, fitness and functional ability will be associated with higher levels of cognitive performance in high functioning independent- living, older South Africans.

## Methods

### Study design and participants

This was a descriptive observational study in which a convenient sample of seventy (n = 70) participants (28 males and 42 females) aged 65 years and older were recruited. Participants were recruited from a commercial gym and from a community-based physical activity intervention program. The Community Health Intervention Programmes (CHIPS) aims to promote physical activity and healthier lifestyles for older adults in marginalized communities in Cape Town [20]. More than a third (39.3%) of Cape Town’s total population lives below the poverty line. Low income households range from no income to earning just over R4166 (304.5$ / 230.3£) per a month. Middle income range from R4166 –R33741.75 (2.462$ / 1.865.2£) per a month and high income above R33741.75 (2.462$ / 1.865.2£) per a month. In addition, 20.8% of the population in the city of Cape Town is unemployed. Our participants were recruited from the Cape flats (Hanover Park and Lotus River) as well as the Southern suburbs (Diep River and Newlands). Half (51.1%) of the individuals living in the Cape flats fall within the low income bracket, 39% within the middle income bracket and 9.9% within the high income bracket. In the Southern suburbs, 30.5% of individuals fall within the low income bracket, 40% within the middle income bracket and 29.6% within the high income bracket. As a result, our participants were older adults who were likely to be physically active and from both high and low socio-economic areas.

Inclusion criteria were that participants had to be older than 65 years, community dwelling and living independently. Those participants who were identified as being at a high risk for coronary artery disease, according to the American College of Sports Medicine criteria [21], required consent from a medical doctor prior to being allowed to complete the test battery.

The exclusion criteria included being previously diagnosed with a stroke, myocardial infarction or uncontrolled disease such as hypertension or diabetes. In addition, those with limited mobility were excluded. Three individuals were excluded as they required the use of assistive walking devices and two individuals due to previously being diagnosed with a mild stroke.

Ethical approval for this research study was obtained from the Research and Ethics Committee of the Faculty of Health Sciences, from the University of Cape Town. Written consent was obtained from each participant before the commencement of the study.

### Measurements

#### Demographic and health status questionnaire

An interviewer-administered questionnaire was administered to obtain information about the participant’s age, occupation, self-perceived health status and highest educational achievement. Participants were also asked to report any previous disease diagnosed by a medical doctor and the associated treatment and medication received.

#### Yale physical activity survey

DiPietro and colleagues developed and validated the Yale Physical Activity Survey (YPAS) for older adults [22] which was later validated in a South African population [23]. The first part of the questionnaire assesses physical activity in five domains, namely; household, yard work, care-giving, exercise and recreational energy expenditure, representing total habitual physical activity. The time spent in each activity was summed to calculate the total physical activity time for the week. Each activity was assigned an intensity code (kcal) as described by DiPietro [22], and subsequently multiplied by the time spent to calculate kcal/week. Each individual’s weekly energy expenditure was calculated for each of the activity domains and then summed to estimate total weekly expenditure.

#### Anthropometry

Height was measured to the nearest centimeter using a wall- mounted tape measure (Detecto). Body mass was measured using a calibrated electric scale (UWB BW-150) and recorded to the nearest 0.5kg. Body mass index (kg/m^2^) was calculated as body mass (kg) divided by height (m) squared [21].

Waist circumference was recorded at the level of the umbilicus and hip circumference was measured at the largest diameter below the umbilicus or maximum circumference over the buttocks. Waist-to-hip ratio was calculated as waist divided by hip circumference [24].

A Harpenden caliper (Holtain) was used to measure skinfold thickness at four sites, namely; sub-scapular, supra-iliac, triceps and biceps. The Durnin and Womersley (1969) equation was used to calculate percentage body fat [25].

### Fitness measures

#### Bicep curls

This test measures upper body muscle strength and endurance [9]. Participants were seated in a chair with back support for posture and given a small dumbbell (2kg’s for females and 4kg’s for males). They were instructed to repeat as many bicep curls as possible in 30 seconds [9].

#### 6—Minute walk test

The participants walked around a 70m indoor track for 6 minutes. Two chairs were placed on the track, one at the start and the other at the 35m mark, in case participants needed to rest during the 6-minute walk. Participants were requested to walk for as long and fast as possible and advised that they could sit and rest if needed. The total distance covered was recorded in metres [26].

### Functional performance tests

#### Static balance test

Static balance was assessed by asking the participant to stand for as long as possible (a maximum of 30 seconds) in the tandem and semi tandem positions, with the left and then right foot in front [27]. The tandem stance was performed by standing with the right foot in front of the left, with the toe of the left foot touching the heel of the right foot, and vice versa with the left foot leading. The time that the stance was maintained without taking a step or losing balance was recorded.

#### Dynamic balance test

The participants were asked to walk 10 meters along a strip of tape using the tandem gait. Tandem gait involves walking heel to toe. When the left foot is in front, the right heel will move forward touching the left toe and when the right foot is in front, the left heel will move forward touching the right toe [28]. The time taken to walk the first 6 steps [27] as well as the time taken to complete 10 meters was recorded.

#### Timed Up and Go test

This test is a measure of mobility, balance and agility. Participants started in a seated position with hands across their chest on a standard chair with no arm rests, then stood up, walked 3 meters, turned around and walked back to the chair and sat down. The time taken to complete the Task was recorded [29].

#### Sit to stand test

This test is a measure of lower body strength. Participants were seated on a standard chair without armrests, with their hands folded across the chest. They were then instructed to rise from the chair into a standing position and sit down again. They repeated this process for 30 seconds without using their arms for assistance. The time taken to complete 5 repetitions was recorded, as well as the total number of sit-to-stands completed in 30 seconds [30].

#### Grip strength

Grip strength was determined using a hand held dynamometer (Takei Physical Fitness Test: Grip-D, T.K.K. 5401) and measured in kilograms (kg) of isometric force. Participants were required to squeeze the hand held dynamometer inner and outer grips towards each other as hard as possible without bending the elbow, while remaining seated [31]. Two attempts each for the right and left arms were recorded.

#### Functional reach

Participants were asked to stand next to a wall and reach as far forward as possible along a wall mounted yardstick (150cm vertically from the ground) without moving, taking a step or lifting their heels [32]. Participants were not allowed to use the wall for support while reaching forward. Each participant was given two familiarization trials and then three performance trials. The mean value (in centimeters) of the last three functional reach trials was recorded.

### Cognitive performance tests

#### The six–item cognitive impairment test (6CIT)

The six–item cognitive impairment test (6CIT) was developed in the United Kingdom to assess cognitive function in older adults by measuring orientation, concentration and short–term memory [33]. This tool was modified for the use in South African older adults [34] and has been validated against the Bristol Activities of Daily Living Scale [35].

The first three questions assess orientation in time, by asking the participant to recall the current month and year. The latter three questions assess memory and concentration. The number of errors made for each question were scored and weighted. The scores range from 0 to 28, where a score from 0 to 7 indicates normal cognitive function, 8 to 9 indicating mild cognitive impairment and 10 to 28 indicating significant cognitive impairment.

#### Modified stroop task

The modified Stroop Task is a computer-based assessment where cues (2cm in height) appear in the centre of a computer monitor [36]. The cues consisted of 4 text words (red, blue, green and yellow) which appeared every 3.2 seconds on a black background and were displayed for 600ms after which a black screen was displayed for 2600ms constituting the response period. The 4 words were presented in 5 different colour inks: red, blue, green, yellow and grey. The words were either presented in grey ink or in a colour ink incongruent with the meaning of the word: e.g. blue word presented in a red colour, or yellow word presented in a green colour, but never a green word presented in a green colour or red word presented in a red colour, etc. The participants were then asked to respond as quickly and as accurately as possible by pressing one of the four response buttons to indicate either the ink colour of the word (if the text was written in either red, blue, green or yellow ink), or the word itself (if the word was written in grey ink).

The Stroop Task measures three executive functions: 1) Shifting between tasks; 2) Updating and monitoring of working memory representation and; 3) Inhibition of dominant or pre-potent responses [37]. The objective of the Stroop Task in this study was to determine the balance between speed and accuracy, which is also known as the speed accuracy trade off [38]. Previous studies have shown that when subjects concentrate more on responding as fast as possible, their number of mistakes increases, thereby sacrificing accuracy for speed [38].

The first Stroop Task administered was regarded as a familiarization trial. The outcome variables such as the number of correct responses, mistakes, missed responses and average reaction times of responses. The average reaction time from the second Stroop Task was used in the data analysis.

## Statistical analysis

STATISTICA 9 software package was used for all the analyses (Stasoft, Inc., Tulsa OK, USA). Descriptive statistics were used to calculate the mean and standard deviations for the demographics, fitness, functional and cognitive results as well as weekly total physical activity time and energy expenditure. Data that were not normally distributed were log transformed. Therefore the author log transformed the following variables: Dynamic balance, 6 Item cognitive impairment test, total mistakes and missed responses on the Stroop test. T-tests were used to determine any significant differences between men and women for anthropometrical measures. Spearman’s correlation coefficients were applied to investigate the relationship between total physical activity and measures of cognitive function. Pearson’s correlation coefficient was used to investigate the relationship between fitness, functional and cognitive performance. In addition, a multiple regression analysis was performed to determine which of the fitness, functional and cognitive performance tests had the greatest influence on cognitive performance. A backwards elimination multiple regression analysis was performed to develop a model to best predict the number of correct responses on the Stroop Task. The backward elimination process started with variables that were significant in the bivariate correlation. Variables were added to the model to determine the amount of variance. Variables that gave the most statistically insignificant deterioration of the model fit were deleted. This process was repeated until no further variables could be deleted without a statistically significant loss of fit.

## Results

### Participant characteristics

A total of 70 participants, 65 years to 84 years, volunteered to participate in the research. Comparisons between males and females for body composition, total physical activity time and energy expenditure are shown in Table 1. Fourteen participants were employed during the time of the study. Twenty eight participants (40%) completed up to primary school, ten participants (14.3%) completed secondary school and thirty two participants (45.7%) completed tertiary education. The most common medical diagnosis was hypertension with thirty two participants (45.7%) being diagnosed, followed by seventeen participants (24.3%) with elevated serum cholesterol concentrations, eight participants (11.4%) with diabetes mellitus, and seven participants (10%) with asthma. A total of 47 participants (67%) were using medication for the various conditions and 18.6% were diagnosed with two or more medical conditions.

**Table 1:** Participant characteristics (data are represented as mean ± standard deviation).

	Total (n = 70)	Men (n = 28)	Women (n = 42)
Age (Years)	70.9 ±4.5	71.0 ±4.2	70.9 ±4.7
Body Mass Index (kg.m^2^)	27.6 ±4.2	27.5 ±3.8	27.8 ±4.4
Waist Circumference (cm)	92.8 ±11.8*	97.5 ±10.0	89.8 ±12.0
Hip Circumference (cm)	106.3 ±9.4	105.0 ±7.2	107.2±10.6
Waist to Hip Ratio	0.9 ±0.1*	0.9 ±0.1	0.8 ±0.1
Percentage Body Fat (%)	29.6 ±4.7*	27.6 ±5.1	31.0 ±4.1
Total Weekly Physical Activity (Minutes)	1450.5 ±850.1	1380.2 ±963.1	1497.3 ±774.4
Total Weekly Energy Expenditure (kcal/week)	5259.6±3143.3	5615.5±3959.7	5022.3±2481.6
*Employment Status*:			
1) Employed (number)	14 (20%)	7 (10%)	7 (10%)
2) Unemployed (number)	56 (80%)	21 (37.5%)	35 (62.5%)
Education Level:			
1) Primary School	28 (40%)	2 (7.1%)	26 (92.9%)
2) Secondary School	10 (14.3%)	5 (50%)	5 (50%)
3) Tertiary Education	32 (45.7%)	21 (65.6%)	11 (34.4%)
Health Status:			
1) Hypertension	32 (45.7%)	12 (37.5%)	20 (62.5)
2) High Cholesterol	17 (24.3%)	7 (41.2%)	10 (58.2%)
3) Diabetes	8 (11.4%)	4 (50%)	4 (50%)
4) Asthma	7 (10%)	5 (71.4%)	2 (28.6%)
5) Peripheral Vascular Disease	1 (1.4%)	1 (1.4%)	0 (0%)

*p< 0.01 –Significant differences between men and women

Employment, Education and Health Status are represented as a number (percentage)

### Fitness, functional and cognitive test results for men and women

The results of the fitness, functional and cognitive tests for men and women are presented in Table 2. The men (599.4m ±118.4) were able to walk significantly further in the 6 minute walk test compared to the women (510.0m ±87.2) with no significant difference in the number of bicep curls in 30 seconds. The mean static balance scores were close to 30 seconds for both men and women. The men (29.7kg ±4.4) also had a significantly higher grip strength score on the left hand compared to the women (19.9kg ±3.7) as well as on the right arm (men: 31.2kg ±5.2 versus women: 20.9kg ±4.5). Grip strength was higher in the right arm for the men compared to the left arm which was also the dominant arm for most of the participants. Functional reach was also significantly different between both groups with the men (86.0cm ±6.2) scoring higher than the women (78.8cm ±5.2). Most of the cognitive performance tests were similar for the men and women with the exception of number of mistakes and correct responses in the Stroop Task. The men (23.4±7.0) had a significantly higher number of correct responses on the Stroop Task compared to the women (16.7±10.0).

**Table 2:** Fitness, functional and cognitive performance for men and women (data are represented as mean ± standard deviation).

	Total (n = 70)	Men (n = 28)	Women (n = 42)
**Physical Fintess:**			
Bicep Curls in 30 seconds (Number)	18.3 ±3.8	19.3 ±3.4	17.7 ±4.0
6 minute walk test (metres)	545.8 ±109.4**	599.4 ±118.4	510.0 ±87.2
**Functional Ability:**			
Static Balance: Tandem Gait: Left Leading (seconds)	27.8 ±6.9	29.1 ±4.6	27.0 ±8.0
Static Balance: Tandem Gait: Right Leading (seconds)	28.5 ±5.5	29.2 ±4.2	28.1 ±6.3
Dynamic Balance: 10 metres	37.2 ±15.3*	30.8 ±16.5	41.7 ±12.8
Timed Up and Go (seconds)	7.2 ±1.5	7.3 ±1.7	7.1 ±1.3
Sit to Stands in 30 seconds (Number)	12.9 ±2.3	13.4 ±2.5	12.5 ±2.1
Grip Strength: Left (kg)	23.8 ±6.3**	29.7 ±4.4	19.9 ±3.7
Right (kg)	25.0 ±7.0**	31.2 ±5.2	20.9 ±4.5
Functional Reach (cm)	81.6 ±6.2**	86.0 ±5.2	78.8 ±5.2
**Cognitive Performance:**			
6 Item Cognitive Impairment Test (errors)	4.0 ±4.1	3.4 ±4.2	4.3 ±4.0
Stroop Task:			
Mistakes (Number)	9.2 ±7.3*	6.3 ±5.1	11.1 ±7.9
Missed Responses (Number)	3.5 ±4.7	2.3 ±4.4	4.3 ±4.9
Correct Responses (Number)	19.3 ±9.5*	23.4 ±7.0	16.7 ±10.0
Average Reaction Time (Seconds)	1585.0 ±373.0	1579.0 ±317.0	1588.0 ±410.5
S.D of Average Reaction time of Correct Responses	454.5 ±175.2	448.0 ±153.1	458.8 ±190.3
Correct Responses: Average Reaction Time (Number/ Second)	0.013 ±0.008*	0.016 ±0.006	0.012 ±0.008

*p< 0.01;

**p< 0.001 –Significant differences between men and women

### Fitness, functional and cognitive test results for young and old older adults

Table 3 represents the fitness, functional and cognitive test results for young older adults and old older adults. There were no significant differences in the fitness and functional variables between both age group classifications. In the cognitive domain, only standard deviation of the average reaction time of correct responses were significantly different between both groups with the young older adult group (411.2ms ±153.8) having a better score than the old older adult group (502.9ms ±187.1).

**Table 3:** Fitness, functional and cognitive performance for young and old older adults (data are represented as mean ± standard deviation).

	Young Older Adults (n = 37) (65–70 years old)	Old Older Adults (n = 33) (70+ years old)
**Physical Fitness:**		
Bicep Curls in 30 seconds (Number)	18.9 ±4.0	17.7 ±3.6
6 minute walk test (metres)	557.1 ±119.5	533.1 ±97.0
**Functional Ability:**		
Static Balance: Tandem Gait: Left Leading (seconds)	28.0 ±6.3	27.7 ±7.5
Static Balance: Tandem Gait: Right Leading (seconds)	29.1 ±4.0	28.0 ±6.9
Dynamic Balance: 10 metres	40.4 ±21.3	41.5 ±21.7
Timed Up and Go (seconds)	6.9 ±1.5	7.4 ±1.4
Sit to Stands in 30 seconds (Number)	13.14 ±2.3	12.6 ±2.2
Grip Strength: Left (kg)	24.7 ±6.6	22.8 ±5.8
Right (kg)	25.6 ±7.1	24.4 ±6.9
Functional Reach (cm)	81.4 ±6.7	82.0 ±5.8
**Cognitive Performance:**		
6 Item Cognitive Impairment Test (errors)	3.6 ±4.0	4.3 ±4.2
Stroop Task:		
Mistakes (Number)	8.2 ±7.0	10.5 ±7.8
Missed Responses (Number)	3.0 ±4.6	3.7 ±4.6
Correct Responses (Number)	20.7 ±9.6	17.8 ±9.2
Average Reaction Time (Seconds)	1535.8 ±417.8	1629.1 ±325.6
S.D of Average Reaction time of Correct Responses	411.2 ±153.8	502.9 ±187.1*
Correct Responses: Average Reaction Time (Number/ Second)	0.014 ±0.01	0.012 ±0.01

*p< 0.01

### Correlations between functional ability and cognitive performance tests

There were significant associations between functional ability and cognitive performance outcomes as illustrated in Table 4. The strongest correlation was between the right arm grip strength and the number of correct responses on the Stroop Task (r = 0.483, p<0.05). In addition there was a significant inverse association between right arm grip strength and the number of errors (r = -0.364, p<0.05). Functional reach was significantly and inversely associated with the number of errors (r = -0.445, p<0.05), and correct responses in the Stroop Task (r = 0.443, p<0.05). The Timed Up and Go test and the sit to stand test did not yield any significant correlations with the cognitive performance components.

**Table 4:** Correlations between functional ability, physical fitness and cognitive performance.

	6 Item cognitive impairment test	Stroop Task					
		Mistakes	Missed Responses	Correct Responses	Average Reaction Time of Correct Responses	S.D of Reaction Time	S.D: Correct Response
**Cognitive Performance Tests**							
6 Item cognitive impairment test		r = 0.468***	r = 0.384***	r = -0.520***	r = 0.060	r = 0.110	r = -0.340*
**Functional Ability Tests**							
Functional Reach	r = -0.367*	r = -0.400*	r = -0.354*	r = 0.443*	r = -0.079	r = 0.005	r = -0.155
Grip Strength: Right arm	r = -0.198	r = -0.364*	r = -0.440*	r = 0.483*	r = -0.105	r = -0.121	r = -0.285*
Dynamic balance	r = 0.074	r = 0.183	r = 0.255*	r = -0.255*	r = 0.036	r = 0.032	r = 0.155
Grip Strength: Left arm	r = -0.177	r = -0.338*	r = -0.393*	r = 0.433*	r = -0.082	r = -0.103	r = -0.191
Timed Up and Go	r = -0.50	r = -0.101	r = -0.073	r = 0.114	r = -0.020	r = -0.066	r = -0.137
Sit to Stands	r = -0.052	r = 0.026	r = 0.021	r = -0.030	r = -0.076	r = -0.096	r = -0.001
Total Physical Activity Time	r = -0.201	r = -0.032	r = -0.180	r = 0.100	r = -0.182	r = -0.205	r = -0.270*
**Physical Fitness Tests**							
Bicep Curls	r = -0.036	r = -0.029	r = -0.139	r = 0.092	r = -0.114	r = -0.132	r = -0.050
6 Minute Walk Test	r = -0.125	r = -0.119	r = -0.285*	r = 0.234	r = 0.017	r = -0.040	r = -0.175

* = p<0.05;

*** = p<0.001;

S.D—Standard Deviation

### Correlations between cognitive performance tests

Associations between the 6-Item Cognitive Impairment test and the Stroop Task variables are illustrated in Table 4. The strongest association was seen between the 6-Item Cognitive Impairment test and the number of correct responses on the Stroop Task (r = 0.520; p<0.001). This suggests that those who perform worse on the 6-Item cognitive impairment test produce fewer correct responses on the Stroop Task. This was also observed between the 6-Item cognitive impairment test and the ratio between standard deviation of the average reaction time of correct responses and the total number of correct responses (r = -0.340; p<0.05)

### Multiple regression analysis to predict the best performance on the novel cognitive stroop task

A backwards elimination multiple regression analysis was performed to develop a model to best predict the number of correct responses on the Stroop Task (Table 5). The final model consisted of four variables including: age (p = 0.053; b = -0.3754), dynamic balance (p = 0.113; b = -0.0700), right arm grip strength (p = 0.002; b = 0.4401) and the score on the 6-item cognitive impairment test (p = 0.000; b = -1.0711) as these variables had strong independent correlations with the number of correct response on the Stroop Task. This model explained 45% of the variance in performance of the Stroop Task. The Timed Up and Go test, sit to stand test, total physical activity time, bicep curls in 30 seconds and 6 minute walk test were not significantly correlated to correct responses on the Stroop task.

**Table 5:** Regression model with correct responses as dependent variable (n = 70).

	b*	Standard Error of b*	b	p value
	Multiple R^2^ = 0.476	Adjusted R^2^ = 0.444		
Intercept			42.079	0.005
Age	-0.178	0.090	-0.375	0.053
Dynamic Balance	-0.157	0.098	-0.070	0.113
Right Grip Strength	0.325	0.100	0.440	0.002*
6 Item Cognitive Impairment Test	-0.464	0.092	-1.071	0.000*

*p < 0.05

Similarly, the ratio between correct responses and average reaction time was explained using the same model. Age (p = 0.032; b = -0.0004), 6-Item cognitive impairment test (p = 0.000; b = -0.0008) and right arm grip strength (p = 0.013; b = 0.0003) contributed significantly to the entire model. The entire model was able to predict 36% of the variance in performance of the Stroop Task ratio between correct response and average reaction.

## Discussion

This study explored relationships between measures of physical fitness, functional ability and cognitive performance in older adults. We hypothesized that a higher level of physical activity, fitness and functional ability would be associated with higher cognitive performance. The data from this study partially supports our hypothesis. We found that functional ability measures such as functional reach, grip strength and dynamic balance were associated with the number of correct responses, mistakes and missed responses on the Stroop Task. Our research has shown that individuals who scored higher on the functional reach test had fewer incorrect responses during the Stroop Task. In addition, we also found a negative association between dynamic balance and the number of correct responses on the Stroop Task. This indicates that those individuals with a faster dynamic balance score had higher correct responses on the Stroop task. Functional reach and dynamic balance are both proxy measures of balance in older adults [28,32]. An individual’s balance is coordinated by a number of physiological systems in the body. A decrease in function of the neuro-musculoskeletal system which helps to maintain balance, may increase the likelihood of falls in older adults [39]. Holtzer et al. 2007 conducted a study to determine the relationship between cognitive function and falls in 127 older adults [40]. Multivariate logistic regressions showed that lower scores on executive attention were associated with the increased risk of recurrent falls in older adults. Similarly, Liu-Ambrose and colleagues conducted a study to determine if executive function is independently associated with balance and mobility in community-dwelling older adults [41]. Results showed that cognitive flexibility was independently associated with balance and mobility in community-dwelling older adults after accounting for age, quadriceps strength and physical activity levels. Therefore, a lack of functional ability may be associated with increased cognitive decline [15]. In addition, another research study by Liu-Ambrose and colleagues have shown improvements in cognitive performance through a resistance and balance training intervention. Liu-Ambrose and colleagues showed the effects of 6 months of resistance and balance training on cognitive function in older adults [42]. Executive function measured by the Stroop task improved significantly in the resistance and balance group when compared to the control group 6 months post intervention.

Another important finding from our study was that right arm grip strength was positively associated with the number of correct responses on the Stroop Task. This may indicate that individuals with greater strength also have better executive function measured by the Stroop task. This finding is in line with the previous work done in the older adult population. Alfaro-Acha et al. 2006, who measured baseline hand grip strength in older adults (n = 2160) and found a significant link between reduced handgrip strength and a decline in cognitive performance over a seven year period [43]. The authors reported that individuals with grip strength in the highest quartile maintained greater levels of cognitive function over the seven-year duration. Handgrip strength is considered to be a simple but important measure of functional ability in older adults and decreases with age due to factors such as a loss in muscle mass [4] (sarcopenia) and strength [44]. As a result, it has been suggested that low grip strength may be an early indicator of subsequent decline in cognitive performance, regardless of education level and gender [43]. Therefore, these particular findings from our study suggests that maintenance of functional ability later in life may not only benefit an individual’s functional capacity, but also play a role in attenuating cognitive decline.

The decline in strength leads to a reduced ability to perform daily tasks. In this regard, Goodpaster and colleagues illustrated that regular participation in moderate physical activity can inhibit both age-related loss of muscle strength as well as increases in muscular fat infiltration in older adults [45]. This was supported by Buchman et al. who concluded that higher levels of physical activity are linked with a slower rate of strength and motor decline in older adults and therefore allow for better quality of life [18]. The maintenance of functional ability over the age of 60 years is important for independence as well as improving activities of daily living such as walking, sitting and change of direction [46]. Therefore, the abovementioned studies suggests that individuals who participate in regular physical activity may be able to carry out functional activities of daily living in a more relaxed and safer manner until later years of life. In addition, this may also lead to older adults being able to take care of themselves and live an independent lifestyle for longer.

In contrast, some of our findings did not support our hypothesis. The Timed Up and Go test, sit to stands, bicep curls in 30 seconds and 6 minute walk test did not yield any significant associations with cognitive performance measures. This is in contrast to previous research which has shown associations between the abovementioned tests and cognitive performance [47,48]. McGough et al. 2011 illustrated a relationship between the Timed Up and Go test and executive function measured by the Trial Making Test part B and the Stroop Word Colour Test in older adults with mild cognitive impairment [48]. These authors concluded that a poorer performance in the Timed Up and Go test was associated with inferior executive function performance on the Trial Making and Stroop Word Colour Test. In another study, Ferreira et al. 2015 illustrated the association between functional performance and executive cognitive functions in older adults which included individuals with low ankle-brachial index [47]. Correlation analysis from the study showed significant associations between the 6 minute walk test and global cognition, working memory, attention span, processing speed and inhibitory control. The 6 minute walk test is often used in older adult research to assess cardiorespiratory fitness. Contrasting results between our study and Ferreira et al. 2015 could be due to our study having a high functioning study sample compared individual’s with low ankle-brachial index which could impact cardiorespiratory fitness. Previous work by Kramer and colleagues have shown that improved cardiorespiratory is positively associated with cognition [49,50] Similarly, other studies have also shown associations between functional ability measured by the bicep curls in 30 seconds test and the sit to stand test and cognitive performance however, this was not shown in our research.

In the present study we also found that habitual levels of physical activity were not associated with grip strength or the Stroop Task variables. This could be due to most of the participants (97%) in our study reported meeting the physical activity guidelines of 150 minutes of moderate intensity exercise for most days of the week, suggesting that they are physically active and highly functioning older adults. This is in contrast to Yaffe et al. who showed that habitual levels of physical activity might partly explain the association with cognitive performance [51]. Yaffe et al. recruited community-dwelling women without cognitive impairment and physical limitations for the study. Those women with higher baseline levels of self-reported physical activity measured by distance walked and total energy expenditure per week were unlikely to experience cognitive decline [51]. Therefore, the amount of physical activity and exercise engaged in by individuals may impact on one’s cognitive performance.

The multiple regression analysis also showed that grip strength, age and results from the 6-item cognitive impairment test explained 45% of the variance in the novel Stroop Task. The number of errors committed was also positively correlated with the 6-item cognitive impairment test. This may be due to a decline of inhibitory processes with ageing, that would lead to a greater error Stroop effect [52] which could promote a delayed response rate, leading an individual to become impulsive thereby creating more errors. Secondly, the stress and anxiety of performing may elicit mistakes as shown by Renaud and Blondin. [53]. This finding may suggest that many attributes are interrelated and may affect more than one domain. Therefore, it could be anticipated that those individuals who are functionally independent may also conserve cognitive function.

We found a significant inverse correlation between the 6-Item cognitive impairment test and the number of correct responses produced on the Stroop Task. Our finding of the relationship between the correct responses on the Stroop Task and scores on the 6-item Cognitive Impairment test is confirmatory though not the primary objective of the study. We also found an inverse correlation between the correct responses: average reaction time ratio and the 6-item cognitive impairment test. Age-related alterations to the central and peripheral nervous systems cause a delay in simple and choice reaction time as well as alterations of one’s capacity to integrate sensory information [5]. Slow reaction time may also be connected to the reduced ability of precise control. Therefore, those individuals who are less cognitively impaired may have a faster reaction time and could be more accurate when responding to the stimuli appearing on the screen during the Stroop Task than individuals with a higher degree of cognitive impairment. Although age related changes occur during an individual’s later years, Grigsby et al concluded that executive functioning is an important determinant of functional status in older adults [54]. In addition, Barnes et al identified cardiovascular physical activity to be positively associated with preservation of cognitive function [55]. Therefore, older adults that are physically active may be able to preserve cognitive function later in life.

### Limitations and strengths

One of the limitations of this research study was that some participants used a computer for the first time during the Stroop Task. This may have impacted on the results obtained, as they may have felt intimidated when completing the test and therefore not given a true indication of cognitive performance. This limitation was addressed by giving each participant one full familiarization trial, and using only the second Stroop Task for data analysis. Another limitation was that the results are based on a measurement taken at a single time point and as a result, we can only determine associations between variables and are only able to theorize the direction of the association. In addition to the limitations mentioned above, our small sample size recruited for the study as well as it being a convenient sample was another limitation to our study.

One of the strengths of this study was that the tests used to assess each domain were well established and recognized. This is also one of few studies investigating cognitive function and functional performance in older adults in low to middle income countries. In addition, the Stroop Task used to measure cognitive function in this study has been widely used and recognized in this research field.

### Summary

In summary, the results of this study showed that measures of physical and functional performance such as grip strength and functional reach were associated with cognitive performance variables on the Stroop Task, even in highly functioning, older, South African adults. However, measures such as the Timed Up and Go, sit to stands, bicep curls in 30 seconds and the 6 minute walk test did not yield any associations. This may suggest that maintenance of one’s functional ability may also impact on cognitive performance later in life thereby allowing a greater independent lifestyle. Future studies should determine the extent to which training improves functional capacity and its impact on cognitive performance.

## Supporting information

S1 File Data PDF. Complete Raw Data PDF for Relationship between Functional Status, Physical Fitness and Cognitive Performance Manuscript.
(PDF)

S2 File Data spreadsheet. Complete Raw Data Spreadsheet for Relationship between Functional Status, Physical Fitness and Cognitive Performance Manuscript.
(XLSX)
