# Supplementary material for: The relationship between functional status, physical fitness and cognitive performance in physically active older adults: A pilot study
Source: PLoS One. 2018 Apr 9;13(4):e0194918. doi: 10.1371/journal.pone.0194918 (PMC5890973; doi:10.1371/journal.pone.0194918)
Supplement: S1 File — Complete Raw Data PDF for Relationship between Functional Status, Physical Fitness and Cognitive Performance Manuscript. (PDF) [file pone.0194918.s001.pdf]

CODE	AGE	AGE GROUPS	SEX	EMPLOYED	JOB.T	MARITAL	YRS ED	HIGH.ED.O
UR001	69	1	1	0	0	1	22	3
UR002	77	2	1	0	0	4	16	3
UR003	65	1	2	1	1	3	14	3
UR004	71	2	1	0	0	2	14	3
UR005	65	1	1	0	0	1	22	3
UR006	66	1	1	0	0	1	24	3
UR007	70	1	2	0	0	1	16	3
UR008	70	1	1	1	1	1	12	2
UR009	68	1	2	0	0	1	12	2
UR010	65	1	2	0	0	1	20	3
UR011	68	1	1	0	0	1	15	3
UR012	68	1	1	0	0	1	16	3
UR013	70	1	1	0	0	1	18	3
UR014	67	1	1	0	0	1	14	3
UR015	72	2	1	0	0	1	23	3
UR016	78	2	2	1	1	3	16	3
UR017	77	2	1	0	0	1	25	3
UR018	74	2	2	1	3	1	7,5	1
UR019	68	1	1	0	0	2	18	3
UR020	81	2	1	0	0	1	18	3
UR021	83	2	2	0	0	1	23	3
UR022	67	1	2	0	0	1	23	3
UR023	70	1	1	0	0	1	17	3
UR024	76	2	1	1	2	1	22	3
UR025	71	2	1	0	0	1	15	3
UR026	78	2	1	0	0	1	20	3
UR027	79	2	2	0	0	4	13	2
UR028	75	2	2	0	0	4	14	2
UR029	84	2	2	0	0	4	15	2
UR030	74	2	1	1	1	1	16	3
UR031	70	1	2	0	0	3	3	1
UR032	65	1	2	0	0	3	7	1
UR033	71	2	2	0	0	1	10	1
UR034	67	1	2	0	0	4	6	1
UR035	73	2	2	0	0	4	8	1
UR036	78	2	2	0	0	4	3	1
UR037	66	1	2	0	0	4	6	1
UR038	71	2	2	0	0	4	8	1
UR039	71	2	2	0	0	4	7	1
UR040	67	1	2	0	0	4	4	1
UR041	69	1	2	0	0	1	8	1
UR042	75	2	2	1	1	1	12	3
UR043	67	1	2	1	3	3	16	3
UR044	67	1	1	0	0	1	12	2
UR045	74	2	1	1	1	1	18	2
UR046	66	1	1	1	4	1	9	1
UR047	66	1	2	1	3	1	10	1
UR048	72	2	1	0	0	1	9	1
UR049	72	2	2	0	0	1	9	1

UR050	70	1	1	0	0	1	17	3
UR051	71	2	2	0	0	1	12	2
UR052	79	2	2	0	0	3	9	1
UR053	66	1	1	0	0	1	16	3
UR054	71	2	1	1	3	1	15	2
UR055	68	1	1	0	0	1	13,5	2
UR056	76	2	1	1	2	1	30	3
UR057	71	2	2	1	2	1	19	3
UR058	68	1	2	0	0	2	8	1
UR059	68	1	2	0	0	2	14	3
UR060	70	1	2	0	0	4	6	1
UR061	68	1	2	0	0	1	6	1
UR062	73	2	2	0	0	4	8	1
UR063	69	1	2	0	0	1	9	1
UR064	68	1	2	0	0	1	7	1
UR065	66	1	2	0	0	2	7	1
UR066	72	2	2	0	0	2	7	1
UR067	69	1	2	0	0	4	11	1
UR068	73	2	2	0	0	4	7	1
UR069	71	2	2	0	0	1	10	1
UR070	69	1	2	0	0	1	19	3

QUALITY H	PERCIEVED	HYPTEN	HI.CHOL	DIABETES	ASTHMA	PVD	TOT.COND:	NONE
5	1	0	0	0	0	0	0	1
3	2	0	0	0	0	0	0	1
4	1	0	1	0	0	0	1	0
4	1	1	0	0	0	1	2	0
5	1	0	0	0	0	0	0	1
4	1	1	1	0	0	0	2	0
4	1	0	0	0	0	0	0	1
4	1	1	0	0	0	0	1	0
4	1	1	0	0	0	0	1	0
5	1	1	1	0	1	0	3	0
5	1	1	1	0	0	0	2	0
5	1	0	0	0	0	0	0	1
5	1	0	0	0	0	0	0	1
4	1	0	1	0	0	0	1	0
5	1	0	1	0	0	0	1	0
4	1	0	0	0	0	0	0	1
3	2	0	0	1	0	0	1	0
5	1	1	0	0	0	0	1	0
4	1	0	1	0	0	0	1	0
5	1	0	0	0	0	0	0	1
3	1	0	1	0	1	0	2	0
5	2	0	0	0	0	0	0	1
3	2	1	0	0	0	0	1	0
4	1	0	0	0	0	0	0	1
4	1	1	0	1	0	0	2	0
4	1	0	0	1	0	0	1	0
3	2	0	1	0	0	0	1	0
4	1	1	0	0	0	0	1	0
4	1	1	0	0	0	0	1	0
4	1	1	1	0	0	0	2	0
3	1	0	0	0	0	0	0	1
5	1	0	0	0	1	0	1	0
5	2	1	0	0	0	0	1	0
4	1	0	0	0	0	0	0	1
4	1	0	0	0	0	0	0	1
3	2	1	0	0	0	0	1	0
3	2	0	0	0	0	0	0	1
3	1	1	0	0	0	0	1	0
4	1	0	1	1	0	0	2	0
3	1	0	0	0	0	0	0	1
4	1	1	1	1	0	0	3	0
4	1	0	1	0	0	0	1	0
4	2	0	0	0	0	0	0	1
4	1	1	0	0	0	0	1	0
5	1	0	0	0	0	0	0	1
3	1	1	1	1	1	0	4	0
4	1	1	0	0	0	0	1	0
4	1	1	0	0	0	0	1	0
3	1	0	0	0	0	0	0	1

4	1	1	0	0	0	0	1	0
4	2	0	0	0	0	0	0	1
4	1	1	1	0	0	0	2	0
4	2	0	0	0	0	0	0	1
4	2	1	0	0	0	0	1	0
3	1	0	0	0	1	0	1	0
4	1	0	0	0	0	0	0	1
5	1	0	0	0	0	0	0	1
3	1	1	1	0	0	0	2	0
4	1	1	0	0	0	0	1	0
3	1	0	0	0	1	0	1	0
3	1	1	0	0	0	0	1	0
3	1	0	0	1	0	0	1	0
3	1	1	0	0	0	0	1	0
3	1	1	0	0	0	0	1	0
3	1	0	0	0	0	0	0	0
3	1	1	0	0	0	0	1	0
3	1	1	1	1	1	0	4	0
3	1	1	0	0	0	0	1	0
3	1	1	0	0	0	0	1	0
5	1	0	0	0	0	0	0	1

MEDs	NO. MEDs	NAME/DOSAGE	HEIGHT	WEIGHT	BMI	WAIST CIR	HIP CIRC
0	0		180	71,8	22,2	90	101,5
1	3	ELTROXIN - 0.15MG ; F	180	100,4	31	115,5	128
1	1	LIPOTAIN	170	59	20,4	85	94,5
1	3	TRI-PLAN - 25MG ; DIS	176	85	27,4	105,5	105
1	1	COXFLAM - 15MG	178	90,5	28,3	109	109
1	3	PHARMA - PRESS - 10M	166	89,3	31,9	109,5	113,5
0	0		162	73	27,9	93	114,5
1	1	PHARMA - PRESS - 10M	169	67,2	23,6	76,75	92,8
1	1	LISORETIC - 20MG	160	66,7	26,1	81,5	109,5
1	3	BURINEX K ; SIMVACOI	156	63,7	26,2	81	102
1	0		157	78,1	31,7	104	107
0	0		180	83,4	25,7	85,5	104
0	0		177,4	71	22,7	92,5	98
1	2	ATACANDT ; CRESTOR	174,5	87,1	27,7	102,5	104,5
1	1	CRESTOR	178	88,3	27,9	98	109
0	0		161	56,4	21,7	76	90
1	2	GLUCOPHAGE ; INSULI	182	93	28,1	101,95	110,15
1	1	DIOVAN - 30MG	167,5	93,6	33,2	108,3	132,25
1	1	FLAXOIL	174	71,3	23,5	85	97
0	0		170,5	70,3	24,1	89,55	96,5
1	1	BUDAFLAME	159,5	54,8	21,4	77	96,45
0	0		164	63,4	23,7	73,5	99,7
1	1	ATTACAND	180	78,6	24,2	88,5	101
0	0		176	87,6	28,3	96	107
1	2	SINOPREN ; GLAMARYI	169	93,7	32,8	110	106,5
1	0		168	66,4	23,5	91	97
1	0		161	55,8	21,5	76	96
1	1	TENORMIN	156,5	56,7	23,3	73	102
1	1	TAREG - 80MG	159	56,8	22,5	84	95
1	1	LINISPARIL 10 - 20MG	173,5	95,7	32	108,5	116
0	0		153,5	72,9	31,1	100,5	121
1	1	ASTHAVENT	148	56,2	25,7	81	101
1	2	RADAQ - 12.5MG ; PH/	155,5	61,8	25,4	83	100,5
0	0		151	61,7	27,1	85,5	104
0	0		150	68,5	30,4	92	113
1	0		147	54,2	25,1	80,5	98,5
0	0		161,4	78,9	30,5	93	111
1	0		156	74,6	30,7	95	114,5
1	0		159,6	85,5	33,4	101	120
0	0		163,2	69	26,1	84	107
1	0		152,5	83,5	36,1	106	120,5
0	0		171	89,1	30,5	100	107
0	0		164	63,6	23,82	84	98
1	1	APPROVAL - 150MG	175	97,1	31,7	105	107,5
0	0		170,1	86,4	29,6	101	108
1	4	PHARMA PRESS, R200(	163	73,4	27,2	91,5	101,6
1	1	TENDOR	155	73,4	30,6	94,2	104,8
1	1	WATER TABLET	179	68,6	21,4	79,45	96,6
0	0		147,5	60,4	27,7	79,35	103,75

1	0		185	89,2	26,2	98	105
0	0		160	65,4	25,2	78	105
1	4	ZILDEN - 60MG; SIMVA	157	71,4	28,6	92	110,75
0	0		171	80,8	27,7	95	104,5
1	1	DISPRIN (HALF)	177	84,8	27,1	102,5	106
1	1	FORIDAL - 200MG POV	155	90	37,5	110	110
0	0		173	70,9	23,7	89	98
0	0		165	52,5	19,3	66	94
1	3	PHARMAPRESS, RIDAC	160,5	97,8	38,2	119	138
1	2	2/DAY	162	76,6	29,2	96	113
1	1	ASTHAVENT	144,5	55,5	26,8	84	98
1	2	HYPACE/ RIDAQ - 1/DA	153,5	64,5	27,2	79,5	105,5
1	2	METFORMEN / GLYCO	150	56,4	25,1	85	96
1	2	HYPACE/ RIDAQ- 1/DA	161	87,1	33,6	111	118
1	2	HYPACE/ RIDAQ - 1/DA	157	67,3	27,4	102	107
0	0		146	62	29,1	86	105,5
1	2	RADAQ (1/DAY)/ AMPI	149,5	69,3	30,8	95	107,5
1	13	PHARMAPRESS / METF	153,5	86,2	36,4	112	125,5
1	2	RIDAQ / PHARMAPRES	148	58,5	26,7	90	97
1	2	RIDAQ / PHARMAPRES	157,2	72,3	29,4	101	116
0	0		158	79	30,9	103	110

W:H	BICEP	TRICEPS	S-ILIAC	SUBSCAP	Total	Body Densi	% BF	B-CURLS
0,8867	12,5	12	7,5	12,5	44,5	1,039539	26,17246	22
0,902344	9,5	22	22,5	19	73	1,022749	33,98988	22
0,899471	5	13,5	6,5	10,5	35,5	1,047346	22,62336	10
1,004762	10,5	17,5	19	25,5	72,5	1,025373	32,75129	22
1	6	18	19	17	60	1,032986	29,19311	24
0,964758	24	17	26,5	8,5	76	1,026113	32,40313	17
0,812227	17	16	19	10	62	1,030059	30,55485	14
0,827047	2,4	2,8	4,1	8,2	17,5	1,064669	14,93341	17
0,744292	7,5	21	15	15	58,5	1,032461	29,43691	17
0,794118	8	19	13,5	17,5	58	1,033914	28,76321	18
0,971963	10	16,5	20	24	70,5	1,027356	31,24876	25
0,822115	4	10	8	15	37	1,044995	23,68634	22
0,943878	5,3	9,5	7,7	10,1	32,6	1,047647	22,48726	22
0,980861	8,3	13,3	19	22	62,6	1,031014	30,10996	25
0,899083	7,1	16	19	19	61,1	1,029647	30,7471	18
0,844444	7,2	18	6	12,8	44	1,036194	27,70955	18
0,925556	4,7	6,7	11,3	14,6	37,3	1,04112	25,44936	16
0,818904	5,4	19,6	12,8	22,2	60	1,029332	30,89419	16
0,876289	3,3	9	6,9	11,6	30,8	1,050013	21,4226	20
0,927979	6,2	8	8,9	11,1	34,2	1,04187	25,1071	14
0,798341	2,1	11,4	10	8,2	31,7	1,043135	24,53098	10
0,737212	3,7	20,3	8,3	8,2	40,5	1,042928	24,62513	19
0,876238	6	12,5	10,4	12,5	41,4	1,041109	25,45455	17
0,897196	8,3	17,2	12,4	18,4	56,3	1,030262	30,46032	20
1,032864	8,2	16,3	17,2	23,2	64,9	1,028403	31,32901	16
0,938144	7,2	17,2	11,4	15,3	51,1	1,032101	29,604	13
0,791667	6,2	19	5	7,6	37,8	1,039944	25,98716	18
0,715686	4,3	17	8	8,3	37,6	1,041713	25,17879	17
0,884211	7	10,7	9	11,5	38,2	1,037626	27,05049	15
0,935345	9,1	24	18,8	23	74,9	1,023264	33,7463	20
0,830579	9,5	17	25,75	30,25	82,5	1,022243	34,2291	16
0,80198	14,5	20,1	18,5	27,9	81	1,024775	33,03265	13
0,825871	10,8	19,5	15	20,8	66,1	1,027901	31,56374	17
0,822115	11,8	20,35	20,8	24,6	77,55	1,025154	32,85412	13
0,814159	9	20,75	19	21	69,75	1,025619	32,6355	20
0,817259	10,4	21,1	13,4	21,4	66,3	1,024977	32,93783	14
0,837838	12,2	19,5	25	26,5	83,2	1,023636	33,57022	18
0,829694	11,7	18,1	23	23,8	76,6	1,023868	33,46095	17
0,841667	18,3	30	26,5	31	105,8	1,015031	37,66964	17
0,785047	8	17,9	11,7	19,7	57,3	1,033434	28,98548	19
0,879668	9,6	19,3	29,7	31,8	90,4	1,020147	35,224	17
0,934579	7,2	19,3	10,5	14	51	1,033373	29,01383	12
0,857143	5,2	10,6	8,7	17,5	42	1,041933	25,0784	19
0,976744	14,35	18,2	22	21	75,55	1,025869	32,51764	21
0,935185	5,7	7,4	6,5	14,4	34	1,044873	23,74186	21
0,900591	8,8	10,8	17,8	28,8	66,2	1,02989	30,63388	14
0,898855	14,7	22,8	16,5	27,6	81,6	1,024168	33,31937	11
0,822464	2,5	3,8	4,9	6,3	17,5	1,063857	15,28827	15
0,764819	10,1	16,5	19,2	18,6	64,4	1,028208	31,42001	12

0,933333	8,4	18,4	11	17	54,8	1,033437	28,98429	20
0,742857	5,5	17,2	16	10,5	49,2	1,03598	27,80836	15
0,8307	15	18,5	28,25	20,25	82	1,018756	35,88684	16
0,909091	5,1	15,8	11,7	19,1	51,7	1,036654	27,49775	16
0,966981	5,4	14,3	9,8	14,3	43,8	1,039161	26,34576	19
1	8	21	17,5	31	77,5	1,024766	33,03711	19
0,908163	3,8	8,3	6,4	15,5	34	1,044061	24,11031	23
0,702128	3	10	5	8,4	26,4	1,053013	20,07969	21
0,862319	14	19,75	18,6	23,75	76,1	1,025265	32,80212	19
0,849558	7,3	16,1	13	19,8	56,2	1,033559	28,92785	27
0,857143	11	21,25	18	20	70,25	1,026641	32,15478	19
0,753555	17,25	22	20	18,5	77,75	1,024678	33,07865	22
0,885417	5,9	16	18,25	21,4	61,55	1,029041	31,03057	23
0,940678	19	21,75	21,5	20,5	82,75	1,022567	34,07604	25
0,953271	25	24,75	21,5	23,5	94,75	1,019268	35,64287	20
0,815166	8,8	18,2	16,5	22,8	66,3	1,029849	30,65315	23
0,883721	21,25	21	18,25	21,5	82	1,021598	34,53514	22
0,89243	19	24,25	17,5	24	84,75	1,021913	34,38556	20
0,927835	14,25	20,75	18	17,5	70,5	1,025326	32,77324	23
0,87069	12,25	15,5	23	25,75	76,5	1,023903	33,44408	22
0,936364	18	37	26	35	116	1,013325	38,49079	19

6 MInWk	TANGAIT LEFT	LOG TANGAIT LEFT	TANGAIT RIGHT	LOG TANGAIT RIGHT	SEM TAN LEFT
785	31	1,491361694	31	1,491361694	30
445	31	1,491361694	31	1,491361694	30
530	31	1,491361694	31	1,491361694	30
700	31	1,491361694	31	1,491361694	30
745	31	1,491361694	31	1,491361694	30
610	31	1,491361694	31	1,491361694	30
495	31	1,491361694	31	1,491361694	30
520	31	1,491361694	31	1,491361694	30
610	25,4	1,404833717	31	1,491361694	30
676	31	1,491361694	31	1,491361694	30
645	31	1,491361694	31	1,491361694	30
786	31	1,491361694	31	1,491361694	30
665	31	1,491361694	31	1,491361694	30
650	31	1,491361694	31	1,491361694	30
672	31	1,491361694	31	1,491361694	30
610	31	1,491361694	31	1,491361694	30
645	31	1,491361694	31	1,491361694	30
552	31	1,491361694	31	1,491361694	30
560	31	1,491361694	31	1,491361694	30
630	31	1,491361694	31	1,491361694	30
530	31	1,491361694	31	1,491361694	30
717	31	1,491361694	31	1,491361694	30
672	31	1,491361694	31	1,491361694	30
618,5	31	1,491361694	31	1,491361694	30
605	31	1,491361694	31	1,491361694	30
560	31	1,491361694	31	1,491361694	30
517	31	1,491361694	31	1,491361694	30
582,5	31	1,491361694	31	1,491361694	30
647,5	31	1,491361694	31	1,491361694	30
597,5	31	1,491361694	31	1,491361694	30
442	6	0,77815125	11	1,041392685	30
525	31	1,491361694	31	1,491361694	30
485	31	1,491361694	15	1,176091259	30
437,5	29,1	1,463892989	31	1,491361694	30
443,3	9	0,954242509	31	1,491361694	30
444	31	1,491361694	31	1,491361694	30
460	31	1,491361694	31	1,491361694	30
432	31	1,491361694	31	1,491361694	30
460	31	1,491361694	31	1,491361694	30
472,5	31	1,491361694	31	1,491361694	30
525	12,5	1,096910013	31	1,491361694	30
532,5	31	1,491361694	31	1,491361694	30
602	31	1,491361694	31	1,491361694	30
734,5	31	1,491361694	31	1,491361694	30
476,5	6,5	0,812913357	8,9	0,949390007	30
307,5	31	1,491361694	31	1,491361694	30
303,5	31	1,491361694	31	1,491361694	30
307,5	31	1,491361694	31	1,491361694	30
306,5	31	1,491361694	31	1,491361694	30

520	31	1,491361694	31	1,491361694	30
510	31	1,491361694	31	1,491361694	30
412,5	1	0	1	0	30
574,4	31	1,491361694	31	1,491361694	30
517,5	31	1,491361694	31	1,491361694	30
570	31	1,491361694	31	1,491361694	30
665	31	1,491361694	31	1,491361694	30
535	31	1,491361694	31	1,491361694	30
414,5	31	1,491361694	31	1,491361694	30
580	31	1,491361694	31	1,491361694	30
539	31	1,491361694	31	1,491361694	30
488	6,8	0,832508913	16,83	1,226084116	30
464	31	1,491361694	31	1,491361694	30
483	31	1,491361694	31	1,491361694	30
516	31	1,491361694	31	1,491361694	30
597,2	31	1,491361694	31	1,491361694	30
525	31	1,491361694	31	1,491361694	30
374	31	1,491361694	31	1,491361694	30
622,5	31	1,491361694	31	1,491361694	30
540,6	31	1,491361694	31	1,491361694	30
482,2	31	1,491361694	31	1,491361694	30

SEM TAN RIGHT	COMP	6 STEPS	10 METRE	LOG 10 METRE	COMPLETE	TIMED UP&GO
30	1	6	24,6	1,390935107	1	7,5
30	1	4	20,7	1,315970345	1	11,7
30	1	7	40	1,602059991	1	8,8
30	1	6,2	26	1,414973348	1	6,9
30	1	6,2	21,8	1,338456494	1	7,1
30	1	6,7	50	1,698970004	1	7,2
30	1	5	26,3	1,419955748	1	8,9
30	1	4,2	20,3	1,307496038	1	9,7
30	0	5,4	31,5	1,498310554	1	7,63
30	1	8,6	43,2	1,635483747	1	7,41
30	1	6,6	37,4	1,572871602	1	6,04
30	1	4,4	21,5	1,33243846	1	5,5
30	1	4,8	25,7	1,409933123	1	6,34
30	1	4,3	24,9	1,396199347	1	4,6
30	1	6,3	37	1,568201724	1	7,6
30	1	7,8	34,1	1,532754379	1	6,1
30	1	3,8	21,7	1,336459734	1	6,5
30	1	11,8	49,5	1,694605199	1	7,56
30	1	3,95	26,52	1,42357352	1	7,7
30	1	5,7	33,9	1,530199698	1	7,8
30	1	8,6	49,4	1,693726949	1	7,2
30	1	4,8	24,17	1,38327665	1	7,42
30	1	7,8	37,4	1,572871602	1	8,49
30	1	5,2	30	1,477121255	1	6,18
30	1	4,2	24,3	1,385606274	1	7,1
30	1	6,2	33,2	1,521138084	1	9,13
30	1	4,2	21,1	1,324282455	1	6,74
30	1	5,8	28	1,447158031	1	6,13
30	1	4,2	24,3	1,385606274	1	7,74
30	1	6	27,5	1,439332694	1	6,6
30	1	7,6	59	1,770852012	1	8,34
30	1	4,7	35,6	1,551449998	1	5,28
30	1	8,1	47	1,672097858	1	7,84
30	0	5,7	48,2	1,683047038	1	8,57
30	1	6	46	1,662757832	1	8,45
30	1	5,6	43,3	1,636487896	1	7,44
30	1	4,8	35	1,544068044	1	6,82
30	1	6	37,9	1,57863921	1	7,62
30	1	6,4	36,5	1,562292864	1	8,17
30	1	6,9	46	1,662757832	1	5,64
30	0	7,1	57	1,755874856	1	5,82
30	1	4,6	25	1,397940009	1	9,42
30	1	4,5	24,3	1,385606274	1	9
30	1	3,9	17,6	1,245512668	1	4,66
30	0	10,2	25,2	1,401400541	0	8,3
30	1	21	104	2,017033339	1	11,23
30	1	13	81	1,908485019	1	8,8
30	1	6,1	34,4	1,536558443	1	7,36
30	1	9	56,2	1,749736316	1	8,87

30	1	5,1	22,8	1,357934847	1	6,84
30	1	5,5	36,2	1,558708571	1	7,2
30	0	5,7	10,8	1,033423755	0	9,08
30	1	4,7	22,5	1,352182518	1	5,9
30	1	6,9	35,7	1,552668216	1	8,93
30	1	3,3	15,9	1,201397124	1	6,74
30	1	7,9	33,5	1,525044807	1	5,35
30	1	8,4	51,4	1,710963119	1	5,51
30	1	6,7	48,1	1,682145076	1	6,5
30	1	12,6	67	1,826074803	1	5,5
30	1	6,5	53,57	1,728921646	1	5,5
30	0	8,3	31,1	1,492760389	0	5,8
30	1	3,8	31	1,491361694	1	5,8
30	1	7	48	1,681241237	1	5,9
30	1	5	28,6	1,456366033	1	5,3
30	1	7,1	49,39	1,693639026	1	5,7
30	1	8,7	33	1,51851394	0	5,2
30	1	8,7	39,76	1,599446376	1	5,6
30	1	11,7	40,57	1,608205008	1	6,3
30	1	8	47	1,672097858	1	6,4
30	1	6,6	34,9	1,542825427	1	7,3

COMPLETE	5 Sit to Stands	SIT TO STAND	COMPLETE	GRIP LEFT	GRIP RIGHT	FR1	FR2
1	8	16	1	28,95	28,75	90	93
1	11	13	1	29,4	34,05	86	87
1	15	10	1	18,35	21,6	89	90
1	13	12	1	30	33,55	92	91
1	9	18	1	26,5	26,5	89	90
1	12	12	1	34,45	30,2	84	85
1	13,3	10	1	19,65	20,35	76	78
1	12,8	13	1	30,4	33,2	80	80
1	12,6	12	1	24,85	27,7	84	84
1	10,2	13	1	19,25	21	76	77
1	9,8	16	1	26,75	31,45	81	82
1	9,8	15	1	32,8	36,5	88	90
1	9,4	16	1	28,65	37,9	90	92
1	8,8	17	1	39,65	36,2	90	90
1	9,1	16	1	34,3	36,45	90	90
1	9,5	14	1	23,05	24,8	83	86
1	12,8	11	1	29,25	31,05	87,7	91,7
1	14,2	10	1	19,25	19,05	82,3	83,5
1	11,67	13	1	34,65	31,95	86	86
1	10,4	13	1	20,55	17,85	82,2	81
1	16,2	9	1	18,8	20,15	80	83
1	11,4	12	1	24,6	25,5	88	86
1	12,5	11	1	29,55	34	87	87
1	10,8	14	1	31,2	35,85	77	78
1	9,7	17	1	29	31,35	85	86
1	12,3	12	1	19,85	23,6	86	85
1	10,2	14	1	12,5	19,65	78	81
1	11,5	14	1	20,75	19,45	80	82
1	11,8	15	1	21	23,45	78	79
1	10,2	13	1	25,25	28,6	93	94
1	13,01	11	1	12,9	14,85	70,2	73,2
1	10,77	14	1	16,8	16,55	75,3	74,8
1	9,8	15	1	21,45	17,65	75,6	75
1	11,7	12	1	13,5	14,55	70	71,4
1	13,62	11	1	11,6	24,4	73	78,2
1	12,51	12	1	19,8	17,85	78,5	76,5
1	12,87	13	1	15	13,3	76,5	77,3
1	10,34	13	1	14,95	18,05	81,4	82,3
1	11,47	13	1	20,6	19,15	81	81,7
1	11,7	13	1	20,55	18,75	84,3	82,7
1	9,3	17	1	24	26,95	70,3	75,1
1	17,9	8	1	27,45	36,7	82	80
1	10	14	1	23,7	25,5	83	83
1	10,9	13	1	28,75	28,15	85	86
1	12,1	12	1	22,25	24,2	78	79
1	23	7	1	28,8	25,45	70	74
1	16,6	10	1	21,55	22,3	65,5	67,8
1	12,7	12	1	33,5	36,6	86,2	88,3
1	16,6	11	1	18,7	19,15	72,2	75,7

1	11,8	12	1	34,55	42,6	92	94
1	11,1	12	1	16,7	16	80	81
1	14,6	9	1	15,75	17,45	81	82
1	11,8	11	1	34,5	31,35	83	82
1	14,8	10	1	29,35	26,8	85	88
1	10,6	13	1	30	30,1	76	76
1	9,16	16	1	28,65	30,3	85	85
1	10,9	13	1	20,85	11,6	84	84
1	13,8	13	1	22	16,8	85	86
1	10,4	14	1	23,7	25	82	82
1	11,6	13	1	22,05	20,6	67	68
1	9,2	16	1	15,9	18,35	78	78
1	11,2	13	1	19,35	19,05	74	69
1	11,74	14	1	19,95	21,9	78	81
1	9,46	16	1	26,15	25	69	75,7
1	12,7	11	1	18,5	18,2	72	72
1	12,4	12	1	23,75	24,75	71	75
1	11,56	14	1	20,5	22,05	76	76
1	9,7	16	1	22,85	24,2	66	66
1	14,9	10	1	20,4	23	79	77
1	13,3	11	1	22,7	24,6	81	82

FR3	AVERAGE	COMPLETE 6CIT		LOG 6CIT	TOTAL PHYSICAL ACT.TIME
91	91,33333	1	7	0,845098	872
87	86,66667	1	1	0	1264
90	89,66667	1	3	0,477121	1240
93	92	1	9	0,954243	1310
90	89,66667	1	3	0,477121	927
87	85,33333	1	1	0	1459
80	78	1	1	0	1453
81	80,33333	1	9	0,954243	2550
85	84,33333	1	1	0	1420
77	76,66667	1	1	0	1740
82	81,66667	1	3	0,477121	1012
90	89,33333	1	3	0,477121	5295
91	91	1	3	0,477121	1560
88	89,33333	1	3	0,477121	1845
90	90	1	3	0,477121	965
87	85,33333	1	5	0,69897	1288
92,7	90,7	1	7	0,845098	2375
83,7	83,16667	1	3	0,477121	2730
87	86,33333	1	1	0	1895
81,7	81,63333	1	1	0	1309
81	81,33333	1	3	0,477121	2105
89	87,66667	1	1	0	1910
88	87,33333	1	1	0	740
80	78,33333	1	15	1,176091	750
86	85,66667	1	1	0	1790
87	86	1	17	1,230449	665
81	80	1	1	0	1900
84	82	1	3	0,477121	1660
80	79	1	3	0,477121	987
93	93,33333	1	1	0	363
72	71,8	1	9	0,954243	400
74,5	74,86667	1	9	0,954243	1205
75,8	75,46667	1	9	0,954243	3505
73,4	71,6	1	17	1,230449	624
78,5	76,56667	1	3	0,477121	1330
81,5	78,83333	1	5	0,69897	670
80,2	78	1	11	1,041393	2270
81,2	81,63333	1	1	0	3330
80	80,9	1	11	1,041393	1350
87,4	84,8	1	7	0,845098	970
76,4	73,93333	1	3	0,477121	1065
82	81,33333	1	7	0,845098	285
84	83,33333	1	1	0	1520
86	85,66667	1	1	0	835
80	79	1	1	0	1155
76	73,33333	1	5	0,69897	729
86,1	73,13333	1	1	0	3190
90,2	88,23333	1	5	0,69897	275
76,8	74,9	1	3	0,477121	1430

94	93,33333	1	1	0	1975
82	81	1	7	0,845098	2550
83	82	1	3	0,477121	1335
83	82,66667	1	3	0,477121	1890
88	87	1	9	0,954243	1310
76	76	1	7	0,845098	1045
85	85	1	3	0,477121	485
85	84,33333	1	1	0	2090
87	86	1	5	0,69897	880
83	82,33333	1	3	0,477121	1830
69	68	1	11	1,041393	615
81	79	1	3	0,477121	1520
76	73	1	11	1,041393	1048
85	81,33333	1	7	0,845098	1485
74,2	72,96667	1	11	1,041393	2345
76	73,33333	1	11	1,041393	785
76	74	1	7	0,845098	1097
77	76,33333	1	3	0,477121	555
69	67	1	7	0,845098	950
78	78	1	10	1	615
81	81,33333	1	1	0	1610

TOTAL ENERGY EXPENDITURE	TOTAL MISTAKES	LOG TOTAL MISTAKES	% TOTAL MISTAKES
3972,5	7	0,84509804	18,75
3799	9	0,954242509	25
3910	7	0,84509804	18,75
5047,5	5	0,698970004	12,5
3981,5	4	0,602059991	9,375
7187,5	6	0,77815125	15,625
3998	2	0,301029996	3,125
12555	20	1,301029996	59,375
4732,5	2	0,301029996	3,125
6390	12	1,079181246	34,375
4689	2	0,301029996	3,125
21250	5	0,698970004	12,5
5790	1	0	0
8110	2	0,301029996	3,125
3502,5	15	1,176091259	43,75
4389	7	0,84509804	18,75
7907,5	9	0,954242509	25
8790	19	1,278753601	56,25
6722,5	5	0,698970004	12,5
5595,5	6	0,77815125	15,625
7247,5	13	1,113943352	37,5
7240	1	0	0
2945	8	0,903089987	21,875
3430	24	1,380211242	71,875
6875	1	0	0
3927,5	8	0,903089987	21,875
3900	17	1,230448921	50
3005	2	0,301029996	3,125
3087	23	1,361727836	68,75
1220,5	8	0,903089987	21,875
1695	24	1,380211242	71,875
3822,5	20	1,301029996	59,375
10942,5	24	1,380211242	71,875
2442,5	16	1,204119983	46,875
5115	3	0,477121255	6,25
2935	17	1,230448921	50
8305	23	1,361727836	68,75
10965	14	1,146128036	40,625
4575	22	1,342422681	65,625
3560	11	1,041392685	31,25
4275	6	0,77815125	15,625
1372,5	2	0,301029996	3,125
5860	3	0,477121255	6,25
3460	11	1,041392685	31,25
5092,5	7	0,84509804	18,75
2497,5	8	0,903089987	21,875
8830	2	0,301029996	3,125
885	5	0,698970004	12,5
4605	4	0,602059991	9,375

6067,5	6	0,77815125	15,625
7770	23	1,361727836	68,75
4827,5	15	1,176091259	43,75
9450	7	0,84509804	18,75
4895	5	0,698970004	12,5
4010	4	0,602059991	9,375
2367,5	7	0,84509804	18,75
6547,5	4	0,602059991	9,375
3370	7	0,84509804	18,75
7840	20	1,301029996	59,375
2545	22	1,342422681	65,625
4737,5	11	1,041392685	31,25
3458	4	0,602059991	9,375
5392,5	12	1,079181246	34,375
9340,5	15	1,176091259	43,75
2905	19	1,278753601	56,25
3724,5	27	1,431363764	81,25
1645	10	1	28,125
3155	21	1,322219295	62,5
2030	9	0,954242509	25
5660	1	0	0

TOTAL MIS.RESP	LOG TOTAL MISSED. RESP	TOTAL CORRECT RESP	% TOTAL CORRECT RESP
1	0	26	81,25
2	0,301029996	23	71,875
1	0	26	81,25
1	0	28	87,5
3	0,477121255	27	84,375
1	0	27	84,375
1	0	31	96,875
3	0,477121255	11	34,375
2	0,301029996	30	93,75
1	0	21	65,625
1	0	31	96,875
4	0,602059991	25	78,125
1	0	32	100
1	0	31	96,875
2	0,301029996	17	53,125
1	0	26	81,25
2	0,301029996	23	71,875
3	0,477121255	12	37,5
1	0	28	87,5
8	0,903089987	20	62,5
3	0,477121255	18	56,25
1	0	32	100
3	0,477121255	23	71,875
4	0,602059991	6	18,75
1	0	32	100
19	1,278753601	7	21,875
5	0,698970004	12	37,5
2	0,301029996	30	93,75
2	0,301029996	9	28,125
1	0	25	78,125
4	0,602059991	6	18,75
9	0,954242509	5	15,625
1	0	9	28,125
6	0,77815125	12	37,5
1	0	30	93,75
11	1,041392685	6	18,75
8	0,903089987	3	9,375
10	1	10	31,25
10	1	2	6,25
10	1	13	40,625
10	1	18	56,25
1	0	31	96,875
1	0	30	93,75
1	0	22	68,75
3	0,477121255	24	75
2	0,301029996	24	75
4	0,602059991	28	87,5
1	0	28	87,5
10	1	20	62,5

1	0	27	84,375
2	0,301029996	9	28,125
8	0,903089987	11	34,375
2	0,301029996	25	78,125
6	0,77815125	23	71,875
2	0,301029996	28	87,5
16	1,204119983	11	34,375
1	0	29	90,625
25	1,397940009	2	6,25
6	0,77815125	8	25
10	1	2	6,25
6	0,77815125	17	53,125
3	0,477121255	27	84,375
1	0	21	65,625
3	0,477121255	16	50
5	0,698970004	10	31,25
2	0,301029996	5	15,625
7	0,84509804	17	53,125
4	0,602059991	9	28,125
8	0,903089987	17	53,125
1	0	32	100

AVE.RT OF ALL CORRECT RESP	S.D OF RT	GREY MISTAKES	GREY MISSED	GREY CORRECT
1658	313	0	0	8
1605	470	1	0	7
1776	482	0	0	8
1610	312	0	0	8
1922	411	1	1	6
1339	432	0	0	8
1148	212	0	0	8
827	131	4	0	4
1830	482	0	1	7
1283	410	3	0	5
1126	268	0	0	8
1706	562	0	0	8
1424	392	0	0	8
1487	294	0	0	8
1575	493	2	1	5
1522	410	2	0	6
1479	515	3	0	5
1676	276	1	2	5
996	205	1	0	7
2164	505	1	2	5
2147	592	0	0	8
1346	302	0	0	8
2087	466	0	0	8
1559	744	4	1	3
1195	383	0	0	8
1780	906	0	7	1
2195	398	4	0	4
1035	356	1	0	7
2121	940	0	0	8
1387	571	2	0	6
1241	247	6	1	1
1249	453	6	2	0
1659	442	2	0	6
2051	614	1	1	6
1085	212	0	0	8
1506	645	5	1	2
878	225	7	1	0
1800	658	1	5	2
1647	99	4	2	2
946	442	2	4	2
2002	411	2	2	4
1266	423	1	0	7
1559	393	1	0	7
1490	484	1	0	7
1834	447	0	0	8
1631	505	3	1	4
969	236	0	0	8
1667	432	0	0	8
1766	567	0	6	2

1402	367	1	0	7
1224	382	1	0	7
1839	701	4	1	3
1920	440	0	0	8
2105	522	0	1	7
1499	399	1	1	6
1731	574	1	5	2
1502	476	0	0	8
1693	920	2	5	1
1247	430	3	1	4
2758	363	4	3	1
1347	508	0	1	7
1612	524	0	0	8
1258	178	2	0	6
1857	555	2	1	5
1976	689	3	1	4
1503	695	3	0	5
2165	556	0	2	6
957	202	1	1	6
2007	725	0	4	4
1730	439	0	0	8

% GREY CORRECT	AVE REC TIME OF CORRECT GREY (NEUTRAL) WORDS	GREY S.D RT
100	1549	306
87,5	1892	471
100	1896	465
100	1626	263
75	2201	506
100	1464	552
100	1158	307
50	918	169
87,5	1776	525
62,5	1287	290
100	1327	250
100	1465	584
100	1277	129
100	1538	221
62,5	1250	643
75	1732	304
62,5	1650	355
62,5	1677	186
87,5	1062	123
62,5	2262	609
100	2080	686
100	1442	377
100	1919	526
37,5	2106	686
100	1492	177
12,5	726	0
50	2291	319
87,5	1277	280
100	2010	940
75	1098	420
12,5	1602	0
0	0	0
75	1712	440
75	1763	377
100	1172	228
25	1735	408
0	0	0
25	1603	158
25	1647	99
25	987	279
50	1922	248
87,5	1550	403
87,5	1363	275
87,5	1773	529
100	1893	412
50	1765	232
100	1151	127
100	1972	340
25	1627	1054

87,5	1786	204
87,5	1226	429
37,5	1482	785
100	1978	450
87,5	2409	557
75	1497	347
25	1998	517
100	1750	616
12,5	2343	0
50	1240	551
12,5	3015	0
87,5	1438	594
100	1615	612
75	1334	148
62,5	1590	267
50	1558	382
62,5	1503	695
75	2483	582
75	941	151
50	2087	1021
100	1997	427

NUMBER OF CORRECT COLOUR-WORD RESPONSES (INCONGRUENT)
18
16
18
20
21
19
23
7
23
16
23
17
24
23
12
20
18
7
21
15
10
24
15
3
24
6
8
23
1
19
5
5
3
6
22
4
3
8
0
11
14
24
23
15
16
20
20
20
18

20
2
8
17
16
22
9
21
1
4
1
10
19
15
11
6
0
11
3
13
24

% COLOUR-WORDS CORRECT RESP (INCONGRUENT)
75
66,66666667
75
83,33333333
87,5
79,16666667
95,83333333
29,16666667
95,83333333
66,66666667
95,83333333
70,83333333
100
95,83333333
50
83,33333333
75
29,16666667
87,5
62,5
41,66666667
100
62,5
12,5
100
25
33,33333333
95,83333333
4,166666667
79,16666667
20,83333333
20,83333333
12,5
25
91,66666667
16,66666667
12,5
33,33333333
0
45,83333333
58,33333333
100
95,83333333
62,5
66,66666667
83,33333333
83,33333333
83,33333333
75

83,33333333
8,333333333
33,33333333
70,83333333
66,66666667
91,66666667
37,5
87,5
4,166666667
16,66666667
4,166666667
41,66666667
79,16666667
62,5
45,83333333
25
0
45,83333333
12,5
54,16666667
100

AVE. REC TIME OF CORRECT COLOUR WORDS (INCONGRUENT)
1707
1480
1722
1604
1843
1287
1144
775
1847
1281
1056
1819
1473
1470
1711
1732
1432
1675
974
2130
2201
1314
2177
1012
1096
1956
2219
961
3008
1478
1169
1249
1553
2340
1053
1392
878
1850
0
939
2025
1183
1619
1358
1805
1605
897
1544
1782

1268
1218
1973
1893
1972
1500
1672
1407
1042
1253
2501
1283
1611
1228
1979
2255
0
1992
989
1982
1642

AVE. REC TIME OF INCORRECT COLOUR -WORDS (INCONGRUENT)
1349
1454
1132
1818
2602
1532
1395
750
1781
981
1206
1160
0
1417
1266
1092
1268
1497
926
2279
2244
0
2470
1079
0
1921
2149
0
1391
1197
1112
1464
1419
1871
808
1458
1105
1393
1787
790
1743
0
1612
1891
1746
2003
908
1910
1477

1501
1318
1617
1974
2377
1540
2084
2086
1675
963
2126
1718
1779
1048
1822
1579
1608
1491
1008
1556
0

AVE. REC TIME OF INCORRECT GREY WORDS (NEUTRAL)	CORR: AVE RT
0	0,015681544
2133	0,014330218
0	0,01463964
0	0,017391304
3197	0,014047867
0	0,020164302
0	0,027003484
852	0,013301088
0	0,016393443
1245	0,016367888
0	0,027531083
0	0,014654162
0	0,02247191
0	0,020847344
1091	0,010793651
1423	0,017082786
843	0,015551048
1103	0,007159905
957	0,02811245
1972	0,009242144
0	0,008383791
0	0,023774146
0	0,011020604
1308	0,003848621
0	0,026778243
0	0,003932584
1845	0,00546697
1406	0,028985507
0	0,004243281
1510	0,018024513
1675	0,004834811
1281	0,004003203
1369	0,005424955
1388	0,005850804
0	0,02764977
1184	0,003984064
825	0,003416856
979	0,005555556
1993	0,001214329
693	0,013742072
2140	0,008991009
1805	0,024486572
1287	0,019243105
1272	0,014765101
0	0,01308615
2204	0,014714899
0	0,028895769
0	0,016796641
0	0,011325028

1115	0,019258203
1966	0,007352941
2327	0,005981512
0	0,013020833
0	0,010926366
2390	0,018679119
855	0,006354708
855	0,01930759
1337	0,001181335
1011	0,006415397
1106	0,000725163
0	0,012620638
0	0,01674938
1249	0,016693164
2186	0,008616047
2202	0,005060729
1967	0,00332668
0	0,007852194
740	0,009404389
0	0,008470354
0	0,01849711

AVE. REC TIME OF ALL MISTAKES
1349
1539
1132
1818
2800
1532
1395
772
1781
1053
1206
1160
0
1417
1241
1202
1709
1475
934
2217
2244
0
2470
1119
0
1921
2073
1406
1391
1286
1259
1406
1415
1838
808
1372
1016
1361
1826
770
1902
1805
1450
1829
1746
2089
908
1910
1477

1424
1347
1820
1974
2377
1823
1879
2086
1562
970
1932
1718
1779
1085
1874
1683
1650
1491
994
1556
0
